# Supplementary material for: The Effects of Chloride Flux on Drosophila Heart Rate
Source: Methods Protoc. 2019 Aug 22;2(3):73. doi: 10.3390/mps2030073 (PMC6789470; doi:10.3390/mps2030073)
Supplement: Supplementary file 1 [file mps-02-00073-s001.pdf]

Article

The Effects of Chloride Flux on *Drosophila* Heart Rate

Catherine E. Stanley [1], Alex S. Mauss [2], Alexander Borst [2] and Robin L. Cooper [1,*]

[1] Department of Biology, Center for Muscle Biology. University of Kentucky, Lexington, KY 40506-0225, USA
[2] Max Planck Institute of Neurobiology, 82152 Martinsried, Germany
* Correspondence: RLCOOP1@uky.edu

Video S1. A representative heart tube ceasing to beat within an intact larvae with blue light exposure in a GtACR2 ^{VK00005} (TM6B) larva. Available online: https://youtu.be/KL0gqq2yDrs

Video S2. A representative heart tube ceasing to beat within an intact larvae yellow light exposure for a halorhodopsin (eNpHR)-expressing larva. In the S2 video, the very bright light stops the heart which is stated in the caption text. With dimmer yellow light one can see the heart rate is slowly beating and when the light is removed the rate picks up drastically. Available online: https://youtu.be/vuNTfQvjOxU

Video S3. A representative heart tube ceasing to beat within an in-situ larval preparation with blue light exposure in a GtACR2 ^{VK00005} (TM6B) larva. Available online: https://youtu.be/RgahJOONGdg

Video S4. A representative heart tube ceasing to beat within an in-situ larval preparation with yellow light exposure for a halorhodopsin-expressing larva. Available online: https://youtu.be/q3Wu1dpJMnY

Video S5. Visual fibrillation of very small contractions along the heart tube for GtACR1^{VK00005} × 24B which was treated with 5-HT (1 μM) to increase the heart rate and exposed to a gradually increasing intense green light (166.5 W/m^2). Available online: https://youtu.be/5sem2mFXB-E

Video S6. A representative GtACR2^{VK00005} × 24B larva illustrating the effect of a dim white light changing to a more intense white light (0.425 W/m^2) and dim blue to high intensity blue light (101.3 W/m^2) in which fibrillation also occurs. Available online: https://youtu.be/3BlC7WEDgGE
